# Triqler for MaxQuant: Enhancing Results from MaxQuant by Bayesian Error Propagation and Integration

**DOI:** 10.1021/acs.jproteome.0c00902

**Published:** 2021-03-04

**Authors:** Matthew The, Lukas Käll

**Affiliations:** † Chair of Proteomics and Bioanalytics, Technische Universität München, Emil-Erlenmeyer Forum 5, 85354 Freising, Germany; ‡ Science for Life Laboratory, School of Engineering Sciences in Chemistry, Biotechnology and Health, Royal Institute of Technology − KTH, Box 1031, 17121 Solna, Sweden

**Keywords:** mass spectrometry, proteomics, Bayesian statistics, quantification, label-free quantification

## Abstract

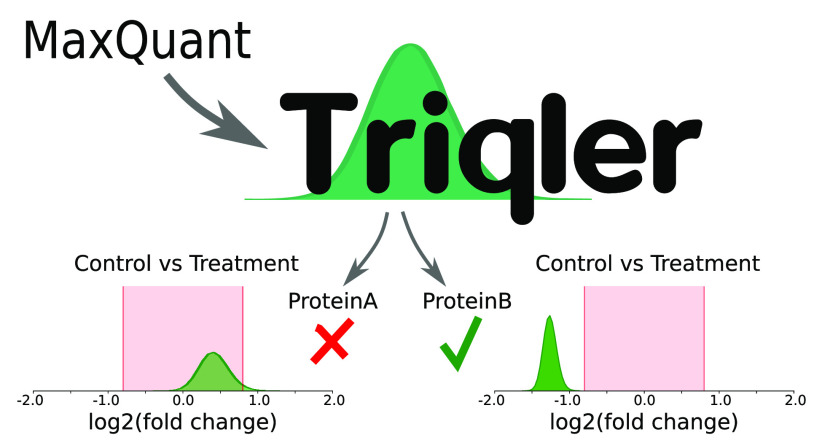

Error estimation for differential protein quantification by label-free shotgun proteomics is challenging due to the multitude of error sources, each contributing uncertainty to the final results. We have previously designed a Bayesian model, Triqler, to combine such error terms into one combined quantification error. Here we present an interface for Triqler that takes MaxQuant results as input, allowing quick reanalysis of already processed data. We demonstrate that Triqler outperforms the original processing for a large set of both engineered and clinical/biological relevant data sets. Triqler and its interface to MaxQuant are available as a Python module under an Apache 2.0 license from https://pypi.org/project/triqler/.

## Introduction

In mass-spectrometry (MS)-based proteomics, label-free quantification (LFQ) enables the study of relative concentrations of proteins in complex mixtures. The technique scales easily to large sample cohorts and can handle complex experimental designs.

The data processing of such samples contains a chain of processing steps that depend on each other. Typically, MS1 features are extracted for each MS run and are subsequently matched to peptide sequences independently of the other runs. Optionally, the different MS runs are aligned to each other, and missing peptide intensities are extracted using so-called match-between-runs techniques.^1,4,18^ In each processing step, thresholds are applied to the measurements based on quality metrics, either statistically motivated or simply selected according to best practices; that is, the data that are deemed reliable are shrinking in each processing step.

Strict partitioning is not just unnecessary, it is also harmful to the performance of LFQ experiments. We previously designed a Bayesian method, Triqler, to integrate different error sources into combined quantification posterior error probabilities, which we demonstrated to dramatically improve both the precision and the recall of LFQ experiments.^12^ It also addressed the issue of missing values, another major problem for LFQ data analysis,^6,15^ by using the Bayesian framework to appropriately assign probabilities to a range of expression values. The advantages of Bayesian statistics in protein quantification is by no means limited to LFQ processing. More generally, it has been successfully applied to isobaric labeling experiments^8,10^ and for clustering quantification data.^13^

Triqler’s lack of support for match-between-runs was addressed in a follow-up article.^13^ However, we are aware that a complete reanalysis using a novel method is not always practical. A very popular pipeline for processing LFQ data is the MaxLFQ^4^ software, which includes match-between-runs, followed by postprocessing with the Perseus package.^14^ We hence find it worthwhile to plug into this user base and provide an alternative means to interpret the output from the MaxLFQ pipeline. We specifically note the potential of the increasingly more common practice of depositing results of processing analyses to repositories, such as MassIVE^3^ and PRIDE.^9^

Both MaxLFQ and Perseus, to large extents, follow the classical threshold-based processing pipeline. Here we show how Triqler can be used in the place of Perseus, which effectively converts the MaxLFQ workflow into a Bayesian processing method. The input to Triqler will in this case be the evidence.txt file directly obtainable from MaxLFQ. The processing not only renders a dramatic performance improvement but also gives a more detailed insight into the reliability of each protein’s quantitative values using Triqler’s new posterior distribution plotting capabilities.

## Methods

### Data Sets

To examine the validity of the results, we analyzed three engineered data sets, where known proteins were spiked into a background at known concentrations. For this, we downloaded MaxQuant result files for a study where UPS proteins were spiked in at three concentrations in a yeast background, which we will refer to as the UPS-Yeast set (PRIDE project: PXD002370, Ratio2_txt.zip and Ratio2.5_txt.zip). We also downloaded MaxQuant evidence.txt files from MassIVE.quant for a reanalysis of the iPRG2015 data set (RMSV000000248.33), in which six proteins were spiked in at four different concentrations in a yeast background, and the MaxLFQ benchmark data set (RMSV000000255.1), with UPS1/UPS2 proteins spiked into an *E. coli* lysate.

Furthermore, we downloaded the MaxQuant results uploaded to PRIDE for four recent biological and clinical studies: (1) a study investigating temozolomide resistance in glioblastoma^17^ (PXD007759), (2) a clinical study examining CD4+ and CD8+ T-cells of multiple sclerosis patients^2^ (PXD011785), (3) a clinical data set investigating biomarkers for cholangiocarcinoma^5^ (PXD011804), and (4) a proteogenomics study of nonsmall and small lung carcinoma cell lines^16^ (PXD015270). For the Glioblastoma data set, as was done in the original study, we only analyzed the results of the samples in the T_ZHI sample group, i.e., the samples treated with temozolomide together with dimethyl sulfoxide, and D_ZHI sample group, i.e., the sample group with dimethyl sulfoxide only, but still used the identifications transferred by match-between-runs reported in the evidene.txt file.

### Data Analysis

MaxQuant evidence.txt files were converted to Triqler input files using the triqler.convert.maxquant program from Triqler v0.6.1. For this, we used the Andromeda scores reported by MaxQuant to compute the Triqler peptide-spectrum match (PSM) score as log(*score*). We used the leading protein(s) as reported by MaxQuant as the corresponding protein for a peptide. Feature intensities were normalized using a retention-time-dependent normalization scheme.^18^ For the Glioblastoma data set, we used the --use_gene_names option to use gene names instead of protein identifiers to allow better comparison with the results from the original study.

The converted input file was then processed by Triqler v0.6.1 with default parameters, except for the minimum number of present values per peptide *S* (--min_samples) and the log_2_ fold-change threshold *F* (--fold_change_eval), which are listed in Table 1 and were chosen based on comparable parameters used in the original studies.

**Table 1:** Summary of Data Sets and Results^a^

data set	samples	groups	*S*	*F*	*DE* proteins (5% FDR)
iPRG2015	12	4	7	0.5	30 tp (max: 30) + 0 fp
MaxLFQ benchmark	8	2	4	1.0	37 tp (max: 40) + 2 fp
UPS-Yeast Ratio2	6	2	3	0.8	9 tp (max: 48) + 0 fp
UPS-Yeast Ratio2.5	6	2	3	0.8	39 tp (max: 48) + 0 fp
Glioblastoma	6	2	3	1.0	270
Multiple sclerosis	27	2	17	0.5	10
Cholangiocarcinoma	30	3	8	0.5	50
Lung cancer	12	2	6	1.0	278

a Results for the engineered data sets (iPRG2015, MaxLFQ benchmark, UPS-Yeast Ratio2, and UPS-Yeast Ratio2.5) demonstrate the high sensitivity and correct FDR control of Triqler. For each of the biological data sets (Glioblastoma, Multiple sclerosis, Cholangiocarcinoma, and Lung cancer), Triqler finds differentially abundant proteins after multiple testing corrections, which the original studies generally were unable to do. *S* is the minimum number of nonmissing values for a peptide to be retained. *F* is the log_2_ fold-change threshold used to evaluate the differential abundance. *DE* proteins is the number of differentially abundant proteins at 5% differential abundance FDR. If more than two groups were present, then this column lists the sum of the differentially abundant proteins for each pairwise comparison. For the engineered data sets, the first number is the number of true-positives (tp), with the maximum number of true-positives given in parentheses, and the last number is the number of false-positives (fp).

### Limitations and Requirements

The data sets presented here used the default MaxLFQ false discovery rate (FDR) threshold of 1%; however, when processing new data sets, we recommend that the FDR thresholds are changed to 100% FDR before starting the MaxQuant processing. This assures that all peptide quantification values are properly transferred to Triqler. Also, although all data sets made use MaxLFQ’s match-between-runs feature, the current converter does not take into account errors from this process.^7^ A better alternative to this is presented by our method, Quandenser,^13^ which evaluates the uncertainty in the feature alignments and thereby increases the precision in the processing even further.

An assumption in the Triqler model is that the majority of the proteins will not change between conditions. However, even if this assumption is violated, we have previously obtained reasonable results.^12^ Furthermore, care should be taken in specifying the maximum allowed number of missing values per peptide. The more missing values are allowed, the less reliable the estimation of the missing value distribution becomes. However, we have observed that Triqler can obtain reasonable error estimates when allowing up to 70% of the runs to have missing values for a peptide.^13^

A typical run of Triqler takes under 5 min and requires <1 GB of RAM. Thus far, we have not observed a case in which Triqler was unable to handle a data set due to too large of a number of PSMs, peptides, or proteins. The largest data set analyzed by Triqler to date had 500 000 PSMs, 60 000 peptides, and 6000 proteins and finished in 10 min using four cores and 4.5 GB of RAM.

## Results and Discussion

We implemented an interface from MaxQuant to Triqler. The interface had only one input, converting MaxQuant evidence.txt to Triqler input files, and required no intervention in the setup of the MaxLFQ processing. We benchmarked the performance of the combination of MaxLFQ and Triqler on all data sets. An overview of the results is given in Table 1, and we will walk through the results for each data set in the sections below.

### Engineered Data Sets

**Figure 1:**
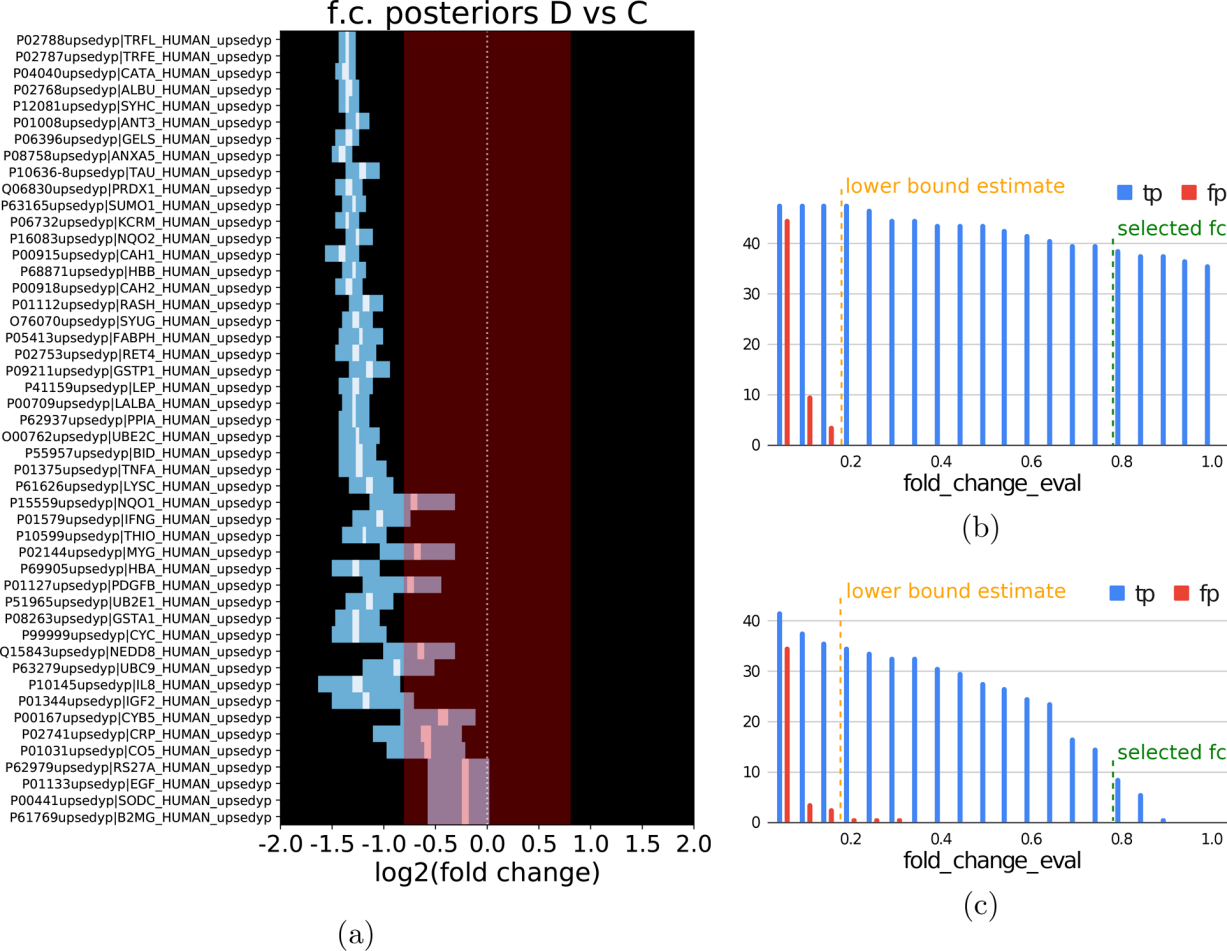
Posterior fold-change distributions allow for a quick and intuitive interpretation of Triqler’s results. (a) Posterior distributions of the log_2_ fold change for the spiked-in UPS proteins in the UPS-Yeast Ratio2.5 data set correctly center around log_2_(2.5) = 1.3. The proteins are sorted by the confidence of the protein identification, with high-confidence proteins (multiple high-confidence peptides) at the top and low-confidence proteins (few or low-confidence peptides) at the bottom. (b) The number of true-positive differentially abundant proteins in the UPS-Yeast Ratio2.5 data set slowly decreases as a function of an increasing fold-change evaluation threshold. The lower bound for this threshold is given in orange and is calculated from the standard deviation of the protein prior distribution. Below this lower bound, the number of false-positives rapidly increases. (c) For the UPS-Yeast Ratio2 set, the initially chosen threshold of 0.8 leads to very low sensitivity. On the basis of the lower bound estimation, a threshold of 0.5 is still within the range where few false-positives will occur.

**Figure 2:**
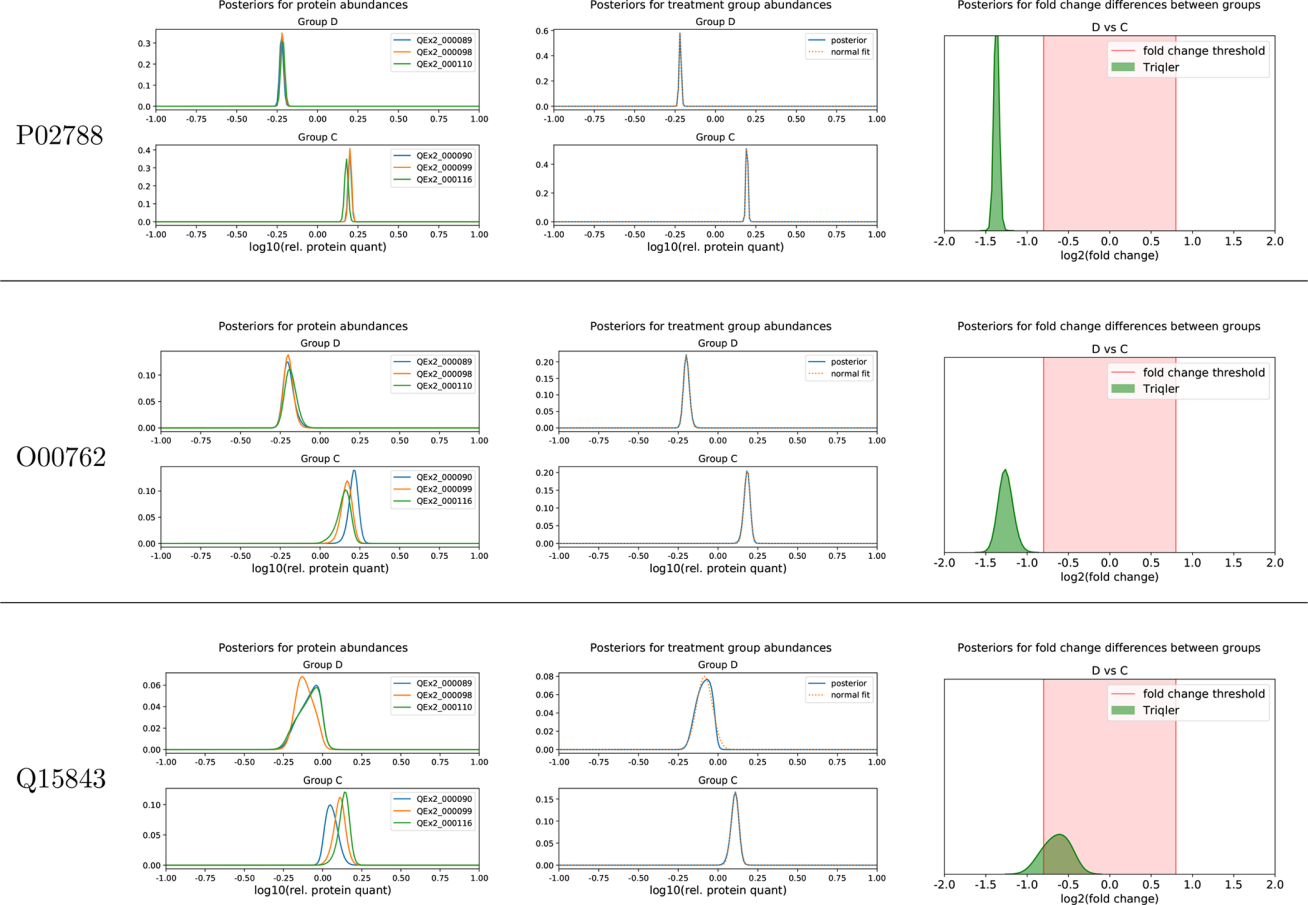
Posterior distributions reflect the uncertainty of the input data. Posterior distributions for three UPS proteins at the protein, the treatment group, and the fold change between group levels for the UPS-Yeast Ratio2.5 data set. The plots exemplify the different degrees of confidence in the differential abundance, as inferred by Triqler. For P02788, we have multiple peptide identifications that all agree on the relative abundances, which leads to a narrow posterior distribution. For O00762 and Q15483, fewer peptides were identified, and some missing values were present, which leads to wider posterior distributions and, in the case of Q15483, a visible influence of the protein prior to “pulling” the distribution toward 0.

First, we characterized the behavior of our new MaxLFQ+Triqler pipeline on three engineered data sets, starting with the UPS-Yeast data set. Employing the fold-change evaluation threshold of 0.8, as in the original Triqler article, we found a comparable performance to the original Triqler pipeline with 39 true-positives and 0 false-positives for the Ratio2.5 set (Figures 1A,B and 2); however, we noted a rather low sensitivity for the Ratio2 set, with only 9 true-positives and 0 false-positives. This seemed to be due to an underestimation of the fold change for the UPS proteins, which Triqler estimated to be closer to the evaluation threshold of 0.8 than to the spike-in ratio of 1.0 (Figure 1C). Lowering the fold-change evaluation threshold to 0.5 appreciably increased the sensitivity to 44 and 28 true-positives for the Ratio2.5 and Ratio2 sets, respectively, while retaining specificity, with 0 false-positives in both cases.

**Figure 3:**
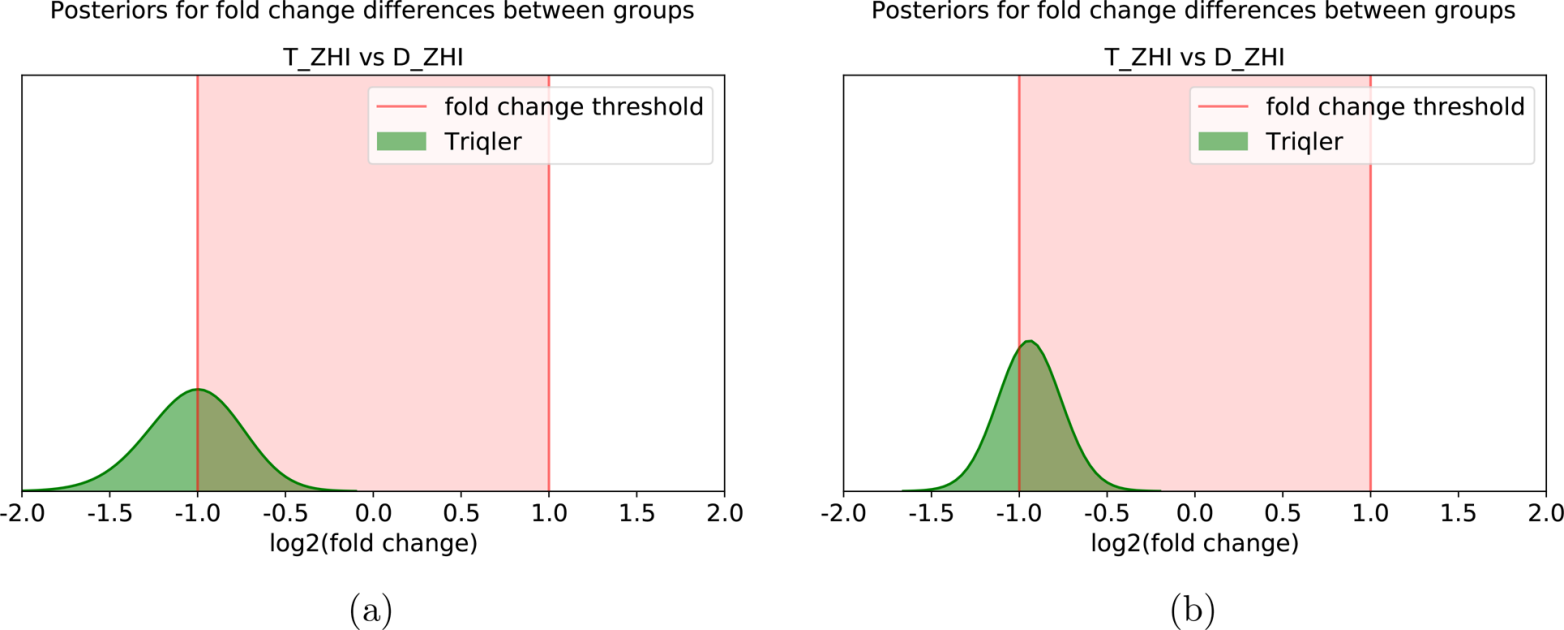
Genes and proteins close to a fold-change threshold risk being overlooked. (a) Gene RPL21 was called differentially expressed in the original study of the Glioblastoma data set but missed the 5% FDR cutoff in the Triqler analysis because the log_2_ fold change was close to 1.0. (b) Gene RPL13 was not called differentially abundant in the original study or by Triqler; however, it shows equally strong evidence of differential expression as RPL21 and should ideally be taken into account as evidence of the regulation of the Ribosome KEGG pathway in downstream pathway analysis tools.

More generally, the fold-change evaluation threshold should be chosen based on the biological question. In practice, many differential expression analyses already use this type of fold-change threshold to filter out low effect sizes. However, there are some differences between this fold-change threshold and our Bayesian fold-change evaluation threshold. First, the Bayesian threshold should be higher than two tot three standard deviations of the fold-change distribution that results from the prior distributions (Supporting Information). Triqler calculates this value from the hyperparameter estimations, prints it in the logs, and warns the user if the chosen threshold is below this value. In practice, this typically leads to a lower bound for the log_2_ fold change of ∼0.5. Below this lower bound, we expect an accumulation of false-positives due to technical or biological variation (Figure 1B,C, Figure S1). Second, for the traditional fold-change cutoff, only the fold change of the group means needs to exceed the chosen value. In Triqler’s case, the bulk of the posterior’s probability mass, for example, 95% to obtain a posterior error probability of 0.05, needs to exceed the chosen value, which is a much more strict requirement. This effect can be seen in Figure 3.

Next, we analyzed the iPRG2015 data set with Triqler using the MaxQuant results from a reanalysis by MaxQuant with match-between-runs uploaded to MassIVE. Our new pipeline resulted in perfect recall and precision at 5% FDR, that is, neither false-positives nor false-negatives. We analyzed this same set as that in The and Käll^12^ with several pipelines without match-between-runs. In that benchmark, the MaxQuant+Perseus pipeline displayed a very low sensitivity, frequently only calling one of the six spike-in proteins as differentially expressed. Furthermore, it showed problems with specificity as well, producing a couple of false-positives. On the contrary, a pipeline using Triqler without MaxQuant identified the spiked-in proteins without any false-positives or false-negatives as well.

Finally, we looked at a MaxQuant reanalysis of the MaxLFQ benchmark data set uploaded to MassIVE. We found 37 out of the 40 differentially abundant UPS proteins at 5% FDR. We also found two of the eight nondifferentially abundant UPS proteins making the 5% FDR cutoff as well, although they were both very close to the FDR threshold.

### Biological and Clinical Data Sets

In the original study of the Glioblastoma data set, the authors found 65 up- and 96 down-regulated genes significantly differentially expressed based on a *t* test and filtering for uncorrected *p* values below 0.05. Unfortunately, the submitted data were not detailed enough to allow the application of multiple testing correction. Triqler found 106 up- and 164 down-regulated genes at 5% FDR. The overlap was 55 genes (25 up- and 30 down-regulated genes). Triqler did not call a large part of the genes found in the original study differentially expressed due to two reasons. First, about half of these genes have only one or two peptides, the evidence of which was not strong enough to overcome the prior (Figure S2). Second, genes with a log_2_ fold changes close to 1.0 will result in posterior fold-change distributions with approximately equal probabilities on both sides of this cutoff (Figure 3a). This highlights the limitations of setting thresholds before the pathway analysis.

**Figure 4:**
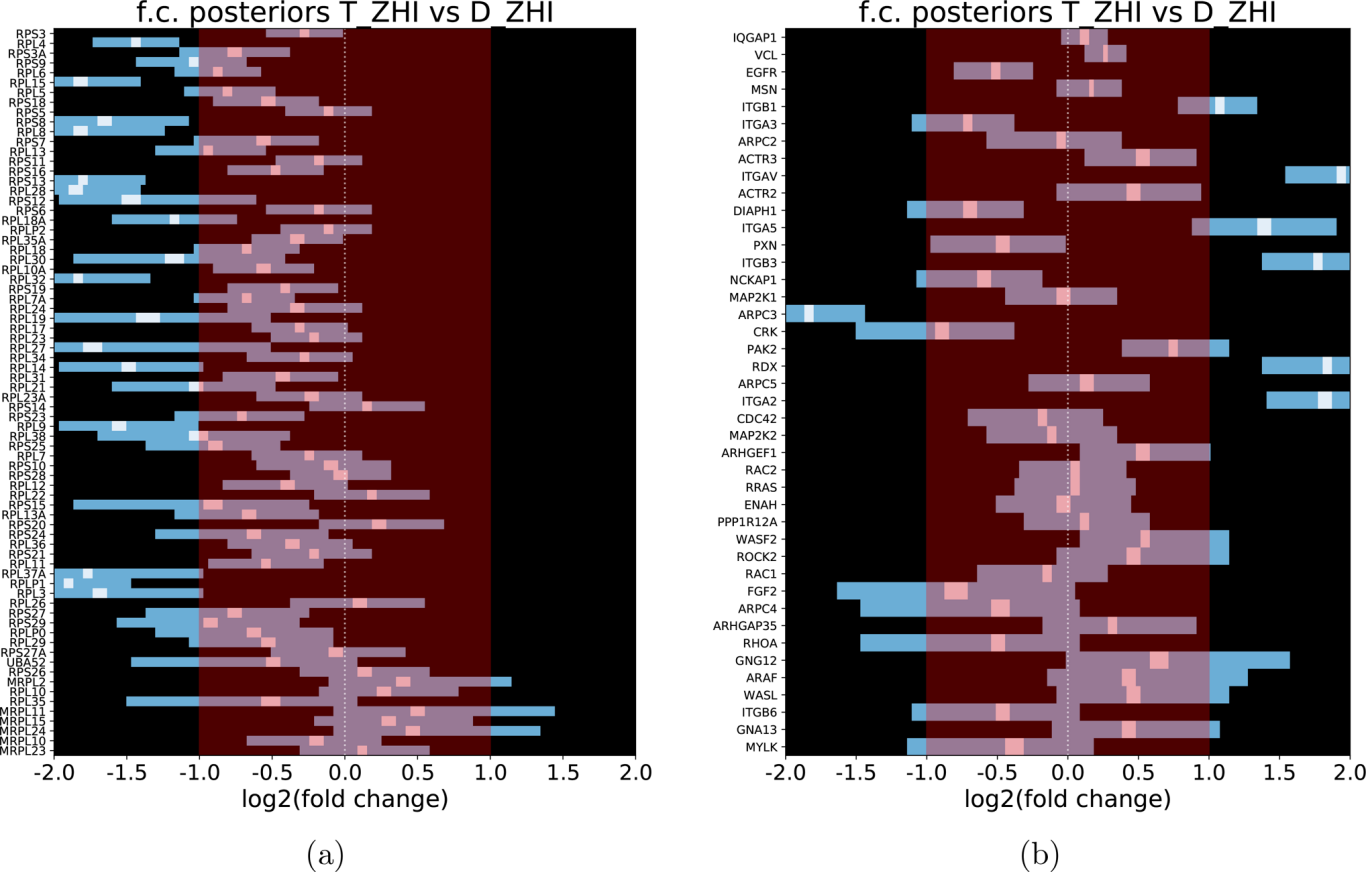
Pathways can be easily inspected by heatmaps of posterior distributions. Heatmap of posterior distributions of the fold change for the (a) Ribosome KEGG pathway (ko03010) and (b) Regulation of actin cytoskeleton KEGG pathway (ko04810) of the Glioblastoma data set. The genes are sorted by confidence of the gene being identified, with genes with multiple high-confidence peptides closer to the top and genes with few or low-confidence peptides toward the bottom. The ko03010 pathway shows very consistent down-regulation, whereas the ko04810 pathway displays both up- and down-regulated genes.

Looking at the most confidently up- and down-regulated KEGG pathways reported in the original study again underlines the issue of using hard partitioning by using a threshold (Figure 4). For the Ribosome pathway (ko03010), many genes show expression values close to a log_2_ fold change of 1.0, for example, RPL13 (Figure 3b). This gene was not reported as differentially expressed in the original study but does show a clear down-regulation in the posterior distribution plot. Triqler allows the user to easily create these heatmap posterior plots based on a list of genes or proteins to get a more thorough image of the pathway expression.

Second, we reprocessed the Multiple sclerosis data set. The original study^2^ reported 228 and 195 differentially abundant proteins for the CD4+ and CD8+ T cells, respectively, using a *p* < 0.05 criterion. After we applied the Benjamini–Hochberg correction for multiple testing, none of these proteins remained significant at 5% differential-abundance FDR. Applying Triqler to MaxQuant’s evidence file resulted in 10 differentially abundant proteins at 5% differential abundance FDR for the CD4+ T cells, with 5 overlapping with the original list of 228 proteins at *p* < 0.05. Most of the proteins called significant at *p* < 0.05 in the original study were not called by Triqler because they were artifacts of the multiple testing problem or were due to low effect size (Figure S3). For the CD8+ T cells, no differentially abundant proteins were found with Triqler at 5% differential abundance FDR, but five proteins were found at 10% differential abundance FDR. None of these appeared in the *p*-value-filtered list of the original study. This appears to have been a result of one of the control samples clustering with the Multiple sclerosis samples, as was also observed in the original study. Triqler’s Bayesian model assigns less importance to such outliers compared with a *t* test.

Next, we investigated the Cholangiocarcinoma data set. Here he original study used a pipeline of MaxLFQ followed by the mixOmics R package.^11^ This resulted in zero, two, and three differentially abundant proteins at 5% differential abundance FDR for the premalignant periductal fibrosis (PDF)–Normal, Cholangiocarcinoma (CCA)–Normal, and CCA–PDF comparisons, respectively. Reanalysis with Triqler resulted in 1, 19, and 30 differentially expressed proteins for these respective comparisons at the same FDR threshold. The sets found by Triqler included the differentially abundant proteins from the original study at 5% FDR, except for Q96PD5, and showed a large overlap with the list of differentially abundant proteins with uncorrected *p* values below 0.05 in the original study (2, 29, and 30 proteins, respectively).

Finally, we reanalyzed the Lung cancer data set. In the original study,^16^ the authors found 147 differentially abundant proteins using |log_2_*FC*| > 1.0 as the criterion without applying any statistical tests. Reanalysis with Triqler resulted in 278 differentially abundant proteins at 5% differential abundance FDR with an overlap of 88 proteins with the list of differentially abundant proteins from the original study. Out of the 14 differentially abundant proteins that had a Pearson correlation >0.4 with microarray mRNA expression levels in the original study, 11 were also called differentially abundant by Triqler.

## Conclusions

Here we have shown that with very little effort, users can extract new information from previously processed data from MaxQuant using our Triqler interface. There is often a sizable overlap with the differentially abundant proteins at *p*-value thresholds, but Triqler is able to eliminate false-positives caused by multiple testing, unreliable data, incoherent data, and poor missing value imputation. Also, instead of reporting three separate values for the identification probability, fold change, and significance value for differential expression for each protein, Triqler reports a posterior fold-change distribution that is intuitive to interpret and incorporates all three pieces of information.

Bayesian statistics is also helpful for downstream analysis. Whereas we here described how to generate statistics for lists of differentially abundant proteins, this is seldom the end goal of an experiment. Often, we strive to examine higher level questions, such as is a particular metabolic pathway differentially regulated, or are the proteins from a certain organelle differentially regulated? When using frequentist tests, we would, in such situations, again partition our findings based on an arbitrary selected FDR threshold; however, at least in theory, this is better done by Bayesian models, allowing uncertainties to propagate to the final question we want to answer.

## Supporting Information Available

Note S1: Influence of the fold-change evaluation threshold. Figure S1: Lower bound estimate for the fold-change evaluation threshold controls the number of false-positives. Table S1: UPS-Yeast Ratio2.5: Number of false-positive and -negative quantifications for different fold-change boundaries. Table S2: UPS-Yeast Ratio2: Number of false -positive and -negative quantifications for different fold-change boundaries. Table S3: MaxLFQ benchmark: Number of true-positive and false-positive and -negative quantifications for different fold-change boundaries. Table S4: iPRG2015: Number of true- and false-positive quantifications for different fold-change boundaries. Table S5: Glioblastoma: Number of significant differential abundant genes for different fold-change boundaries. Table S6: Multiple sclerosis CD4: Number of significant differential abundant proteins for different fold-change boundaries. Table S7: Multiple sclerosis CD8: Number of significant differential abundant proteins for different fold-change boundaries. Table S8: Cholangiocarcinoma: Number of significant differential abundant proteins for different fold-change boundaries. Table S9: Lung cancer: Number of significant differential abundant proteins for different fold-change boundaries. Figure S2: Posterior distributions of the fold-change difference for up- and down-regulated genes according to the original study of the Glioblastoma data set. Figure S3: Posterior distributions of the fold-change difference for up- and down-regulated genes according to the original study of the CD4+ and CD8+ data sets (PDF)

## Notes

Triqler and its interface to MaxQuant are available as a Python module under an Apache 2.0 license from https://pypi.org/project/triqler/.
