# Supplementary material for: Triqler for MaxQuant: Enhancing Results from MaxQuant by Bayesian Error Propagation and Integration
Source: J Proteome Res. 2021 Mar 4;20(4):2062–8. doi: 10.1021/acs.jproteome.0c00902 (PMC8041382; doi:10.1021/acs.jproteome.0c00902)
Supplement: Supplementary file 1 — pr0c00902_si_001.pdf [file pr0c00902_si_001.pdf]

Supplement to: "Triqler for MaxQuant: Enhancing results from MaxQuant by Bayesian error propagation and integration"

Matthew The[1] and Lukas Käll[2,*]

[1]Chair of Proteomics and Bioanalytics, Technische Universität München, Emil-Erlenmeyer Forum 5, 85354 Freising, Germany
[2]Science for Life Laboratory, School of Engineering Sciences in Chemistry, Biotechnology and Health, Royal Institute of Technology – KTH, Box 1031, 17121 Solna, Sweden
[*]Corresponding author: lukask@kth.se

February 16, 2021

Note S1: Influence of the fold change evaluation threshold

We varied the fold change evaluation threshold `--fold_change_eval` over a range of values to show how the number of significant proteins or genes depends on it. For the engineered datasets, we could show that we obtain a high number of true positives for a large range of values without losing control of the prescribed 5% FDR, as long as we stay above the lower bound estimate given by (Supplementary Figure S1):

$$L = \frac{\log_2 (10^{\sigma_y}) \cdot \sqrt{2}}{\sqrt{N}} \cdot 2.5, \quad (1)$$

where σ_y is the standard deviation of the protein prior in $\log_{10}$ abundance units, N is the number of samples in the group, the $\sqrt{2}$ corrects for the fact that we subtract 2 normal distributions to calculate the fold change distribution and the factor 2.5 ensures that 99% of the probability distribution of the normal distribution is contained.

We verified empirically that this lower bound estimate ensures that 99% of the fold change posterior distribution is below this threshold when only very weak peptide evidence is given for a protein.

In the tables below, we show the results for the number of differentially abundant proteins or genes for both the engineered and biological datasets, with the estimated lower bound marked in orange and the actually used threshold marked in green.

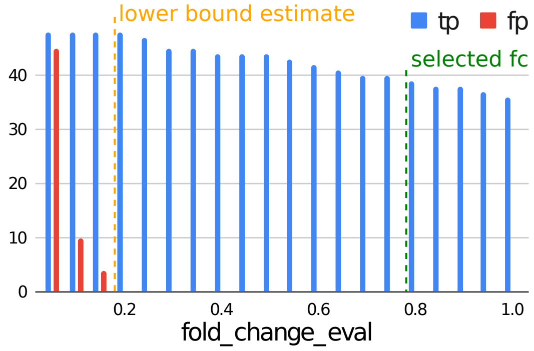

(a) UPS-Yeast Ratio2.5

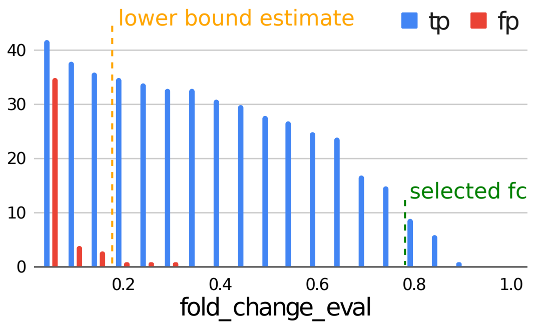

(b) UPS-Yeast Ratio2

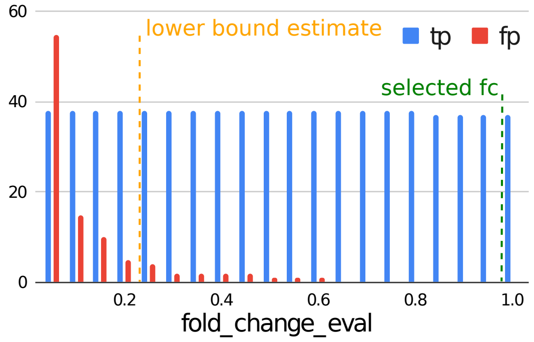

(c) MaxLFQ benchmark

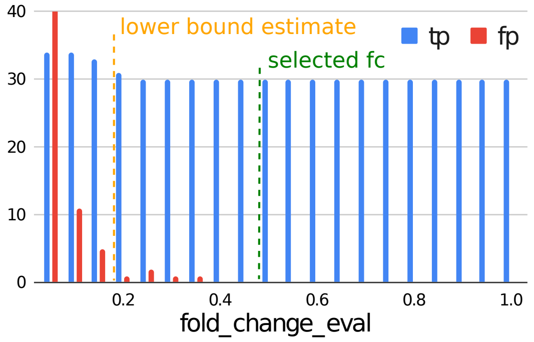

(d) iPRG2015

Figure S1: **The lower bound estimate for the fold change evaluation threshold controls the number of false positives.** Number of true (blue) and false (red) positives at different fold change evaluation thresholds for all engineered datasets. Whereas the selected threshold (green) should be chosen based on the biological question, the lower bound estimate (orange) provides a lower limit of how low this threshold can be set without incurring many false positives.

Table S1: **UPS-Yeast Ratio2.5: Number of false positive and negative quantifications for different fold-change boundaries** The figures for the lower bound estimate of 0.19 is highlighted in yellow, while the selected boundary of 0.8 is marked in green.

FC eval	tp	fp
0.05	48	45
0.1	48	10
0.15	48	4
0.2	48	0
0.25	47	0
0.3	45	0
0.35	45	0
0.4	44	0
0.45	44	0
0.5	44	0
0.55	43	0
0.6	42	0
0.65	41	0
0.7	40	0
0.75	40	0
0.8	39	0
0.85	38	0
0.9	38	0
0.95	37	0
1.0	36	0

Table S2: **UPS-Yeast Ratio2: Number of false positive and negative quantifications for different fold-change boundaries** The figures for the lower bound estimate of 0.19 is highlighted in yellow, while the selected boundary of 0.8 is marked in green.

FC eval	tp	fp
0.05	42	35
0.1	38	4
0.15	36	3
0.2	35	1
0.25	34	1
0.3	33	1
0.35	33	0
0.4	31	0
0.45	30	0
0.5	28	0
0.55	27	0
0.6	25	0
0.65	24	0
0.7	17	0
0.75	15	0
0.8	9	0
0.85	6	0
0.9	1	0
0.95	0	0
1.0	0	0

Table S3: **MaxLFQ benchmark: Number of true positive, false positive and negative quantifications for different fold-change boundaries** The figures for the lower bound estimate of 0.23 is highlighted in yellow, while the selected boundary of 1.0 is marked in green. The majority of the false positives are the UPS proteins that should be present in the same concentrations in UPS1 and UPS2. As can be seen in Figure 6A of the MaxLFQ paper, these proteins are actually differentially abundant.

FC eval	tp	fp	fp*
0.05	38	62	55
0.1	38	22	15
0.15	38	17	10
0.2	38	12	5
0.25	38	11	4
0.3	38	8	2
0.35	38	8	2
0.4	38	8	2
0.45	38	8	2
0.5	38	7	1
0.55	38	7	1
0.6	38	6	1
0.65	38	6	0
0.7	38	4	0
0.75	38	4	0
0.8	38	3	0
0.85	37	3	0
0.9	37	3	0
0.95	37	2	0
1.0	37	2	0

* with equimolar UPS proteins removed

Table S4: **iPRG2015: Number of true and false positive quantifications for different fold-change boundaries** The figures for the lower bound estimate of 0.18 is highlighted in yellow, while the selected boundary of 0.5 is marked in green.

FC eval	1vs2		1vs3		1vs4		2vs3		2vs4		3vs4	
	tp	fp	tp	fp	tp	fp	tp	fp	tp	fp	tp	fp
0.05	6	5	5	32	5	16	6	14	6	13	6	25
0.1	6	0	5	5	5	1	6	1	6	2	6	2
0.15	6	0	4	4	5	0	6	0	6	1	6	0
0.2	5	0	4	1	5	0	6	0	6	0	5	0
0.25	5	0	4	1	5	0	5	1	6	0	5	0
0.3	5	0	4	0	5	0	5	1	6	0	5	0
0.35	5	0	4	0	5	0	5	1	6	0	5	0
0.4	5	0	4	0	5	0	5	0	6	0	5	0
0.45	5	0	4	0	5	0	5	0	6	0	5	0
0.5	5	0	4	0	5	0	5	0	6	0	5	0
0.55	5	0	4	0	5	0	5	0	6	0	5	0
0.6	5	0	4	0	5	0	5	0	6	0	5	0
0.65	5	0	4	0	5	0	5	0	6	0	5	0
0.7	5	0	4	0	5	0	5	0	6	0	5	0
0.75	5	0	4	0	5	0	5	0	6	0	5	0
0.8	5	0	4	0	5	0	5	0	6	0	5	0
0.85	5	0	4	0	5	0	5	0	6	0	5	0
0.9	5	0	4	0	5	0	5	0	6	0	5	0
0.95	5	0	4	0	5	0	5	0	6	0	5	0
1.0	5	0	4	0	5	0	5	0	6	0	5	0

Table S5: **Glioblastoma: Number of significant differential abundant genes for different fold-change boundaries** The figures for the lower bound estimate of 0.51 is highlighted in yellow, while the selected boundary of 1.0 is marked in green.

FC eval	DE genes
0.1	1318
0.2	1001
0.3	806
0.4	672
0.5	568
0.6	478
0.7	412
0.8	361
0.9	311
1.0	270
1.1	238
1.2	207
1.3	181
1.4	161
1.5	142
1.6	124
1.7	107
1.8	91
1.9	81
2.0	71

Table S6: **Multiple Sclerosis CD4: Number of significant differential abundant proteins for different fold-change boundaries** The figures for the lower bound estimate of 0.20 is highlighted in yellow, while the selected boundary of 0.5 is marked in green.

FC eval	DE proteins
0.1	33
0.2	22
0.3	16
0.4	13
0.5	10
0.6	8
0.7	6
0.8	3
0.9	2
1.0	0
1.1	0
1.2	0
1.3	0
1.4	0
1.5	0
1.6	0
1.7	0
1.8	0
1.9	0
2.0	0

Table S7: **Multiple Sclerosis CD8: Number of significant differential abundant proteins for different fold-change boundaries** The figures for the lower bound estimate of 0.20 is highlighted in yellow, while the selected boundary of 0.5 is marked in green.

FC eval	DE proteins
0.1	62
0.2	33
0.3	15
0.4	3
0.5	0
0.6	0
0.7	0
0.8	0
0.9	0
1.0	0
1.1	0
1.2	0
1.3	0
1.4	0
1.5	0
1.6	0
1.7	0
1.8	0
1.9	0
2.0	0

Table S8: **Cholangiocarcinoma: Number of significant differential abundant proteins for different fold-change boundaries** The figures for the lower bound estimate of 0.40 is highlighted in yellow, while the selected boundary of 0.5 is marked in green.

FC eval	CCA vs N	CCA vs PDF	N vs PDF
0.1	92	90	14
0.2	61	58	5
0.3	46	40	4
0.4	37	28	2
0.5	30	19	1
0.6	22	14	1
0.7	16	10	0
0.8	12	7	0
0.9	9	5	0
1.0	7	4	0
1.1	3	3	0
1.2	1	3	0
1.3	0	3	0
1.4	0	2	0
1.5	0	2	0
1.6	0	2	0
1.7	0	1	0
1.8	0	0	0
1.9	0	0	0
2.0	0	0	0

Table S9: **Lung cancer: Number of significant differential abundant proteins for different fold-change boundaries** The figures for the lower bound estimate of 0.54 is highlighted in yellow, while the selected boundary of 1.0 is marked in green.

FC eval	DE proteins
0.1	1519
0.2	1248
0.3	1040
0.4	862
0.5	513
0.6	525
0.7	410
0.8	341
0.9	334
1.0	278
1.1	233
1.2	201
1.3	171
1.4	306
1.5	261
1.6	108
1.7	97
1.8	87
1.9	75
2.0	67

Comparison to original studies

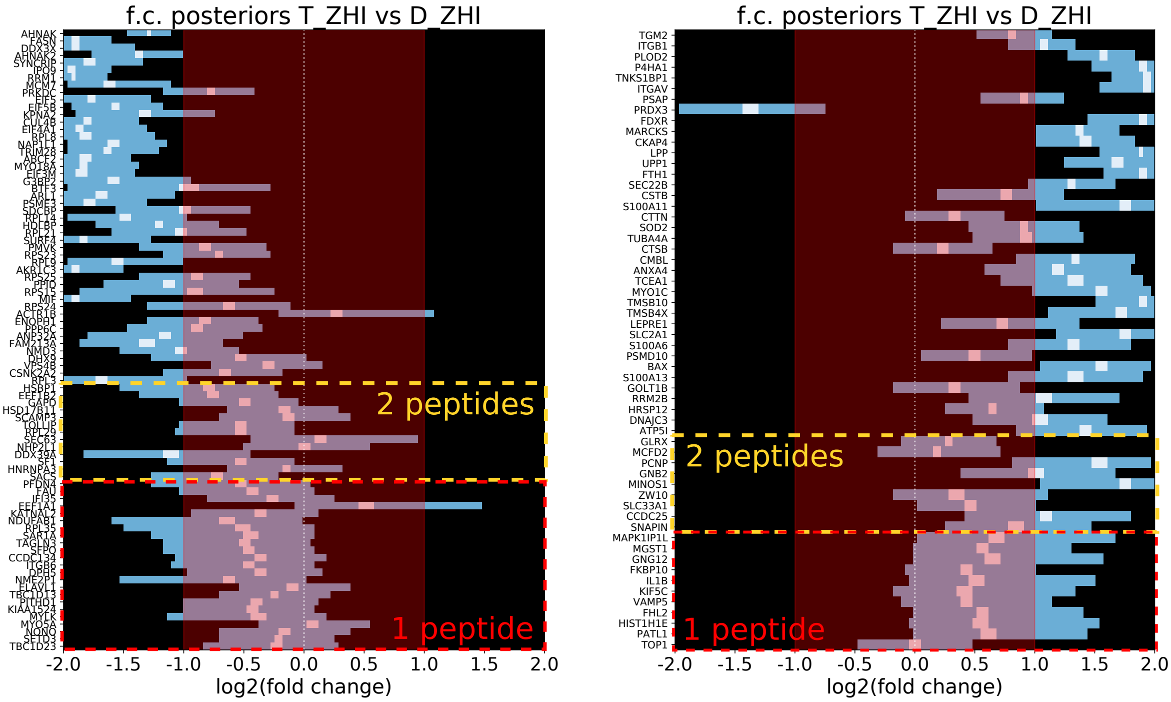

Figure S2: **Triqler curbs one-hit wonders by requiring the evidence to overrule the prior.** Posterior distributions of the fold change difference for down-regulated (left) and up-regulated (right) genes according to the original study of the Glioblastoma dataset using a $p < 0.05$ criterium. The genes are sorted by the confidence of the gene identification, with high-confidence genes (multiple high-confident peptides) at the top and low-confidence genes (few and/or low-confident peptides) at the bottom.

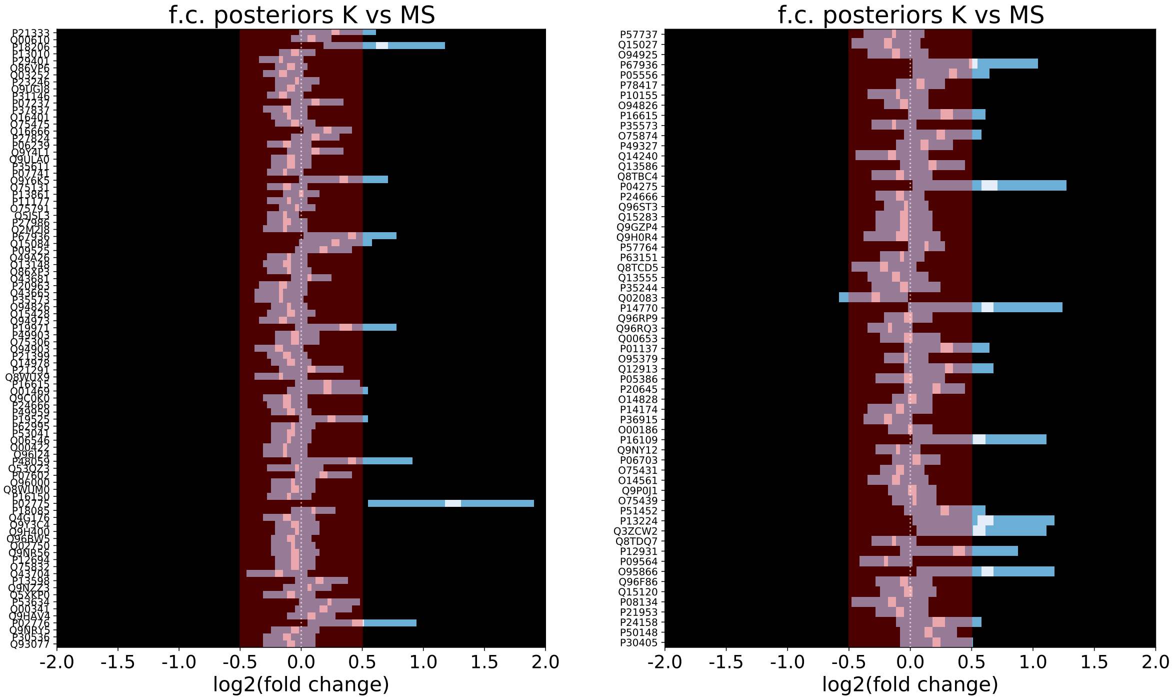

Figure S3: **Triqler addresses problems of multiple testing and low effect sizes.** Posterior distributions of the fold change difference for regulated proteins in the CD4+ (left) and CD8+ (right) comparisons according to the original study of the Multiple sclerosis dataset. The authors used a combined $p < 0.01$, $|log2\_FC| > 0.2$ and $AUC > 0.8$ criterium, where at least two out of the three criteria had to be satisfied. The proteins are sorted by the confidence of the protein identification. After applying multiple testing correction to the list of proteins with $p < 0.05$ from the original study, none of the proteins remained significant at 5% FDR. Triqler can distinguish between proteins with high fold changes with consistent peptide quantification evidence and those that obtain low p-values due to the large number of samples while having low effect sizes.
